# The prebiotic properties of polysaccharides obtained by differentiated deproteinization methods from *Flos Sophorae Immaturus* on *Lactobacillus fermentum*

**DOI:** 10.3389/fmicb.2022.1007267

**Published:** 2022-10-25

**Authors:** Wenting Zhong, Chunmiao Yang, Yongze Zhang, Dongsheng Yang

**Affiliations:** ^1^College of Pharmacy and Food Science, Zhuhai College of Science and Technology, Zhuhai, China; ^2^College of Life Science, Jilin University, Changchun, China

**Keywords:** *Lactobacillus fermentum*, *Flos Sophorae Immaturus*, polysaccharide deproteinization, TCA method, isolation and purification, physiological properties of enzymes

## Abstract

The polysaccharides derived from various deproteinization methods were prepared from *Flos Sophorae Immaturus* (FSI) to investigate the prebiotic efficacy of *Lactobacillus fermentum* (*L.f* ). The implications of polysaccharides from FSI (PFSI) gained after purification performed by non-deproteinization and different deproteinization processes (Savage method, papain method, and TCA method) *via* one-factor optimization were firstly investigated for the influences on the growth of *L.f*. The utilization of carbohydrate sources and the synthesis of protein and lactate during its growth were analyzed, as well as the variations of LDH, SOD, and GSH- Px enzyme dynamics. The results showed that the one-factor optimization of the deproteinization process with the protein removal rate and polysaccharide retention rate as the indexes led to the optimal methods of the Sevage method with 5 elution times, papain method with 80 U/mL concentration, and TCA method with 2.5 ratio, respectively. In addition, the PFSI obtained with or without deproteinization purification had a certain effect on promoting *L.f* proliferation. Moreover, the PFSI gained by the third deproteinization purification, at a concentration of 10 g/L, significantly elevated *L.f* biomass and growth rate compared with the blank control, and the utilization of reducing sugars and the synthesis of protein and lactic acid were higher than the control (P < 0.05); improved LDH, SOD, and GSH-Px activity in *L.f* (P < 0.05), and the TCA method could be effectively applied to eliminate the proteins affecting FSI in probiotics, and PFSI may be a potentially beneficial prebiotic and intestinal reinforcer.

## Introduction

It is widely recognized that Lactobacillus is a group of microorganisms that live in the body to benefit the health of the host and its role in maintaining human health and regulating immune function. As one of the probiotics, Lactobacillus is considered one of the best approved for the use of probiotics in the gastrointestinal tract. This is because it comprises a remarkably high percentage of glycosyltransferases and β-fructosidases, enabling the hydrolysis of prebiotics into smaller fractions and their use as substrates for fermentation. The predominant product of the fermentation of the probiotic strain of bacteria is an organic acid, which provides acidic conditions in the intestine and indirectly inhibits the growth of pathogens. This mode of action confers probiotics to significantly exert the composition of the colonic microbiota in the gastrointestinal tract, thus in turn enhancing host health (Lee et al., 2002; Vergara et al., 2010). A few articles (Yoo et al., 2012; Ajanth Praveen et al., 2019; Seong et al., 2019; Andreani et al., 2021; Dawood et al., 2021; Zhou et al., 2022) confirm that Lactobacillus also has some prebiotic properties. Therefore, how to maximize the value of the symbiotic relationship between prebiotics and probiotics is the focus of current research. There are reports (Ao et al., 2015) that *Lactobacillus fermentum* (*L.f* ) is one of the dominant bacteria in Lactobacillus, which is not only widespread in traditional fermented foods such as kimchi, sauerkraut, and other fermented foods but also its functions and properties are being recognized. As a normal flora in the intestinal tract of animals, *L.f* can stimulate the development of the immune system (Mikelsaar and Zilmer, 2009), modulate the immunity of the body (Bao et al., 2010), strengthen the immune tolerance ability of animals (Li et al., 2011), and other characteristics. However, the probiotic effect of Lactobacillus fermentum has not been thoroughly discussed. Therefore, *L.f* , as a potential probiotic that is extensively present in traditional food, will have great application prospects.

In an environment where viruses are rampant, consumers are gradually becoming more aware of the intentional need for functional foods. Prebiotics has emerged as a food ingredient that benefits the balance of intestinal microorganisms and facilitates the fermentation of bacteria in the intestinal tract, and hence are applied in the food industry for probiotics growth, as well as in the livestock industry as a treatment to enhance the restoration of un-balanced intestinal flora, and regulate metabolic processes in livestock (Mohd et al., 2017). Prebiotics for maintaining intestinal micro-ecological balance would be important, but currently, there are only a few carbohydrate components that qualify as prebiotics. Some of these carbohydrates, such as Fucoidan (Zhu et al., 2021), inulin (Konar et al., 2018), polysaccharides from Chinese Jujube (Yan et al., 2018), and molokhia leaves polysaccharide (Lee et al., 2021), exhibit favorable proliferative properties for probiotic bacteria. Consequently, the exploration of healthy, effective, and low-cost prebiotics is essential for the development of human health.

Saccharides are commonly found in plants that are important biomolecules with comparatively low-grade toxicity and are available for therapeutic purposes or as adjunctive therapeutic agents. *Flos Sophorae Immaturus* (FSI), as one of the homologous Chinese traditional medicines, have polysaccharides (Wu and Zhu, 2018) that are among the activated components to exert pharmacological effects, have confirmed the pharmacological functions of FSI extracts, which are hypoglycemic (Zhang et al., 2017), hypotensive (Li and Li, 2022), antioxidant (Wang F. et al., 2021), anti-aging (Wang et al., 2021), antibacterial and antimicrobial (Zu et al., 2018), and antitumor (Jin et al., 2005). Since FSI belongs to a protein-rich botanical, a large number of the proteins will precipitate along with the procedure of preparing PFSI, thus deproteinization plays an extremely important role in the refinement and constitutive relationship investigation of polysaccharides from FSI (PFSI). *L.f* , introduced in the article, belongs to the genus Lactobacillus, and helps to prevent intestinal diseases and improve immunity and intestinal health. The study was conducted to identify and evaluate the conditions of the protein removal from PFSI by Sevage method, papain method, and trichloroacetic acid (TCA) method, and to observe the impacts on the *in vitro* performance and enzymatic dynamics of *L.f* by incorporating various purification methods of PFSI. In anticipation of supplying the scientific grounds on which to formulate PFSI and *L.f* combinations, it can be regarded as the theoretical reference for future fundamental research on FSI prebiotics, simultaneously serving as a conceptual justification for further separations and purifications of PFSI.

## Materials and methods

### Materials

The materials used for the study include FSI (20210820, Shandong Bozhou R&B Food Sales Co., Ltd., China); *L.f* (GDMCC 1.1796), *Lactobacillus acidophilus* (*L.a*; GDMCC 1.208) and *Lactobacillus plantarum* (*L.p*; GDMCC 1.140) (Guangdong Province Microbial Strain Conservation Center, China); Inulin (food grade) (China Food & Moon Health Technology Co.); Glucose, Papain, Glutathione Peroxidase (GSH-PX) assay kit (Colorimetric method), Superoxide Dismutase (SOD) assay kit (WST-1 method), Lactate dehydrogenase (LDH) assay kit, total protein quantitative assay kit (Thomas Brilliant Blue method) and DNS reagent (Shanghai Yuanye Biotechnology Co., Ltd., China); MRS broth, MRS medium (with agar), and MRS broth (without glucose) (Guangdong Haibo Microbial Technology Co., China).

### Extraction, isolation, and purification of polysaccharides

**Figure 1:**
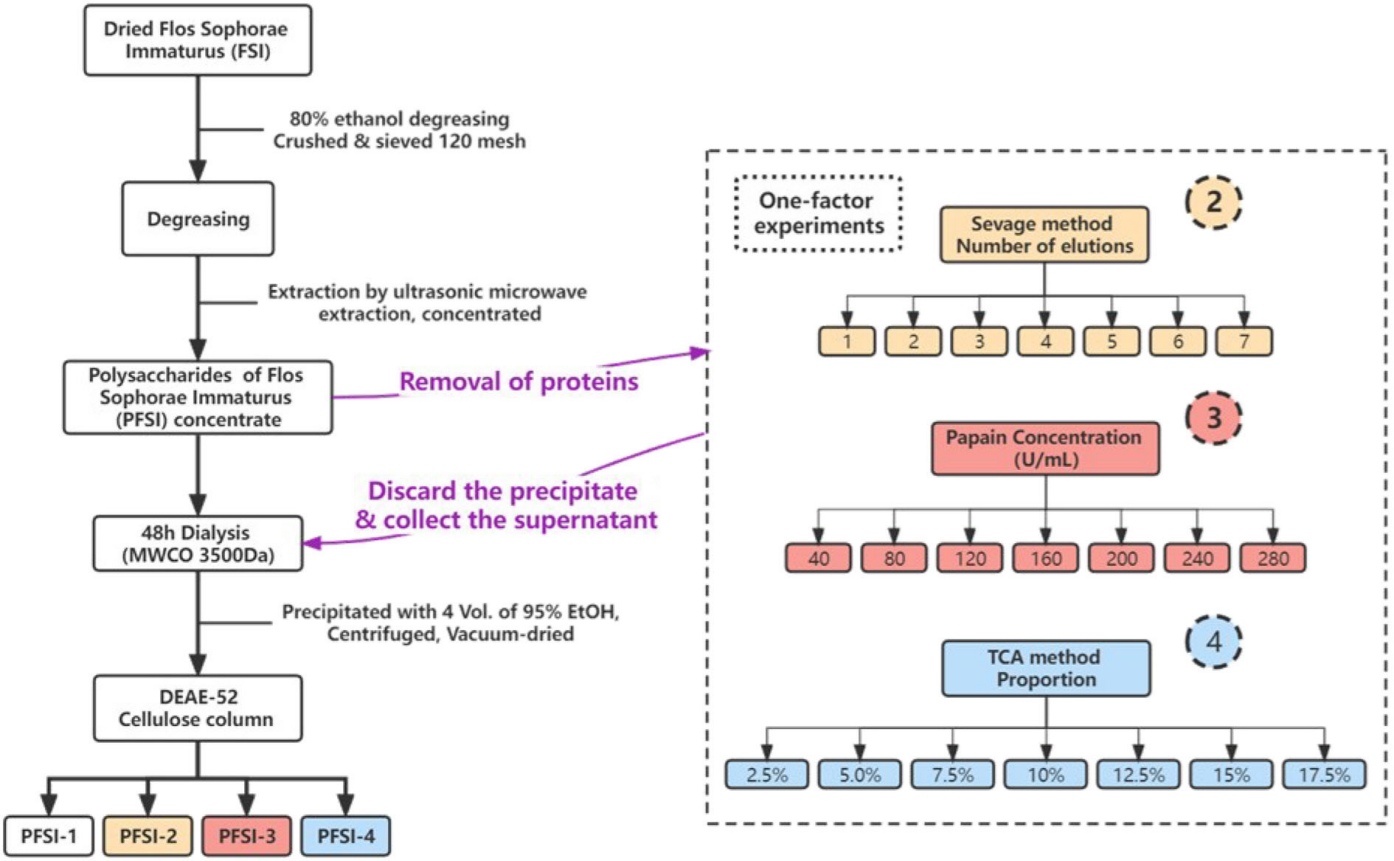
The extraction, isolation, and purification procedure of PFSI. In the figure, circles 2, 3, and 4 represent the distinct deproteinization methods of the Sevage method, papain method, and TCA method, respectively.

An appropriate amount of degreased FSI powder was weighed with 120 mesh and the PFSI was extracted using an ultrasonic-microwave assisted method with the parameters of microwave power 500 W, ultrasonic power 270 W, extraction time 21 min, and material-liquid ratio 1:95. It was cooled at room temperature and centrifuged for 10 min (8,000 rpm/min). The supernatant was transferred and concentrated to obtain the polysaccharide concentrate. The polysaccharide concentrates were then deproteinated using the Sevage method, papain, and TCA method, respectively, and the polysaccharide retention ratio (Y_1_) and protein removal ratio (Y_2_) were analyzed. Ultimately the overall results were evaluated by an overall score (0.5 Y_1_+ 0.5 Y_2_), which was optimized to select the best deproteination process corresponding to various deproteination methods. The polysaccharide concentrates were obtained and placed in molecular weight cut-off (MWCO) 3,500 Da dialysis bags and were treated in running water for 48 h. To the solution of polysaccharide supernatant, 4 times the volume of anhydrous ethanol was added and refrigerated at 4°C overnight. This was followed by washing the lower layer of the precipitate with anhydrous ethanol several times and it was desiccated under decompression. The concentration of the dried products was set at 100 mg/mL and eluted with 200 mL of distilled water, and 0.1, 0.2, and 0.3 mol/L of sodium chloride solutions separately at a filtration rate of 1 mL/min. The solvents were collected, and the polysaccharides were purified, whereas the polysaccharide concentrates without deproteinization were prepared with the above purification operation as PFSI-1. The determination of total saccharide content was conducted by the phenol-sulfuric acid method (Ren et al., 2019). It was calculated that the polysaccharide yield of PFSI-1 after purification was 56.62 ± 1.77%. The determination of protein content was referenced to the instructions of the Kemas Brilliant Blue kit. The extraction, isolation, and purification process of different PFSI are illustrated in Figure 1. The yield of polysaccharides was measured as follows:


$$\begin{array}{l} Y\;\left(\%\right)\;=\;\frac{V\times C\times D}{m\times 10^{3}}\;\times \;100 \end{array}$$

Where Y is the yield of polysaccharide (%), V is the total volume of sample solution (mL), C is the concentration of polysaccharide (mg/mL), D is the dilution factor, and m is the mass of the weighed sample (g).

### Determination of UV spectra

The above polysaccharide sample solution was prepared as 10 mg/mL, and distilled water was used as the control for scanning at a full wavelength within 200 ~ 600 nm.

### Determination of infrared spectra

The fully dried KBr and polysaccharide samples were blended and ground with an agate mortar in a sufficiently dry environment at a mass ratio of 100: 1–200: 1. The slices were pressed and scanned with an infrared spectrometer in the range of 400–4,000 cm^−1^.

### Determination of physicochemical properties of polysaccharides

The PFSI was identified based on the Molisch reaction precipitation method (Pawar et al., 2018) and the 3,5-dinitrosalicylic acid (DNS) method according to the standard procedure reported by Alexander et al. (2011) and McCleary and McGeough (2015).

### Revitalization of *Lactobacillus* sp.

The MRS culture was sterilized at 121°C for 15 min, then the plates were inverted, and the *L.f* (*L.a* or *L.p*) lyophilized powder was inoculated into the MRS modified medium plates in a sterile environment, incubated at 37°C until 36 h. After two generations of incubation, a single *L.f* (*L.a* or *L.p*) colony was picked for two generations of liquid culture, and subsequently, a liquid bacterial suspension was fabricated.

### Effect of different carbohydrate sources on the proliferation of *L.f*

The medium was prepared to contain dissolved polysaccharides at a mass concentration of 2.5 g/L using various purification levels of PFSI, inulin, and glucose, as the carbohydrate source, with ultrasonic-assisted dissolution. After sterilization at 121°C and chilling to ambient temperature, the insoluble substances were removed by filtration through a membrane. The inoculum was 5% 1.5×10^8^ CFU/ml *L.f* suspension and cultivated at 37°C for 36 h. The OD_600_ was measured at 0 h and 36 h. The positive controls were inulin and glucose, and the negative control was the glycose-free medium. The samples were tested three times simultaneously. The biomass was measured by centrifuging the suspension at 3,500 r/min for 10 min, after which the bacteria were gathered and washed twice with phosphate buffer. Using phosphate buffer as a blank control, the absorbance value of the suspension was assessed at 600 nm, which indicated the biomass by absorbance value.

### The comparison of the proliferative effect on *Lactobacillus* sp.

The same process as in 2.7 was adopted. The carbohydrate source for preparing the MRS medium was dissolved by ultrasonication at 1.25, 2.5, 5.0, and 10 g/L to obtain four groups of optimal carbon sources with different concentrations. OD_600_ was measured after 36 h of incubation of *L.f* , *L.a*, and *L.p*. The negative control was a glycose-free MRS medium (concentration of 0 g/L). Each sample was tested three times simultaneously.

### The growth curve of the optimal carbohydrate sources on *L.f*

The same process as in 2.7 was used to attain the optimal carbohydrate source. As the carbohydrate sources for the formulation of MRS medium, ultra-sonication to solubilization, which were 1.25, 2.5, 5.0, and 10 g/L, yielded four groups of different concentrations of the optimum carbohydrate sources. The OD_600_ was measured at 0 h, 6, 12, 18, 24, 30, and 36 h. The negative control group was glycose-free MRS medium. Each sample was tested three times simultaneously.

### The determination of reducing saccharide concentration

The fermentation solutions were cultured in the optimal carbohydrate source at 0 h, 6, 12, 18, 24, 30, and 36 h by centrifugation at 6,000 r/min for 10 min, and then the supernatant fermentation broth was withdrawn and set aside. The method described in McCleary and McGeough (2015) was referred to and modified, the standard curve was plotted, and the regression equation y = 0.8319x−0.0828 was calculated with associated coefficient *R*^2^ = 0.9998. To determine the reducing saccharide content in the fermentation medium using the DNS method, 1 mL of each was taken in the proportion of DNS reagent: the supernatant (V/V) = 1:1, and left to react fully in a boiling water bath for 5 min, taken out and placed in an ice bath to cool down rapidly. The absorbance was measured at 540 nm. Distilled water was applied as a blank control group and the optimum concentration of carbohydrate source of 10 g/L was adopted as the experimental group and the negative control group was glycose-free MRS medium. Each sample was tested three times simultaneously.

### The determination of protein content

The measurement was determined using the kit instructions, by taking 50 uL of supernatant fermentation broth obtained above and kneading it with 3 mL of Kemas Brilliant Blue col-or solution and letting it stand for 10 min to determine the OD value at 595 nm. Distilled water was used as a blank control group and 0.524 g/L of the standard was considered a positive control group. Each sample was tested three times in parallel. The following formula was used for the calculation.


$$\begin{array}{l} Protein\;concentration\;\left(gprot/L\right)\;=\;\frac{\left(OD_{2}\;-\;OD_{0}\right)}{\left(OD_{1}\;-\;OD_{0}\right)}\;\times \;C_{1}\;\times \;D \end{array}$$

In the formula, OD_0_ refers to the absorbance value of the blank group at 595 nm, OD_1_ refers to the absorbance value of the positive control group at 595 nm, OD_2_ refers to the absorbance value of the sample to be measured at 595 nm, C_1_ refers to the concentration of the standard protein, and D refers to the dilution multiple.

### The determination of lactate concentration

The fermentation supernatant was obtained above. The lactate content was determined by the p-hydroxyphenyl method detailed in Meng et al. (2017), and the standard curve was plotted to obtain the regression equation y = 0.0554x−0.0278 with the correlation coefficient *R*^2^ = 0.9997. Each sample was tested three times simultaneously.

### The determination of LDH, SOD, and GSH-Px dynamics

LDH, SOD, and GSH-Px dynamics were measured in the fermentation solution obtained above. Sequentially to the instructions for the experiment, the simultaneous tests were repeated three times.

### Statistical analysis

Data are shown as mean ± SEM, and statistical analysis was performed using Origin 2021, Graphpad Prism 8, and EXCEL 2019. To compare the differences between multiple groups, a one-way analysis of variance (ANOVA) was performed in this software. P < 0.05 indicates statistical significance.

## Results

### The analysis of deproteinization

#### Deproteinization by the Sevage method

**Figure 2:**
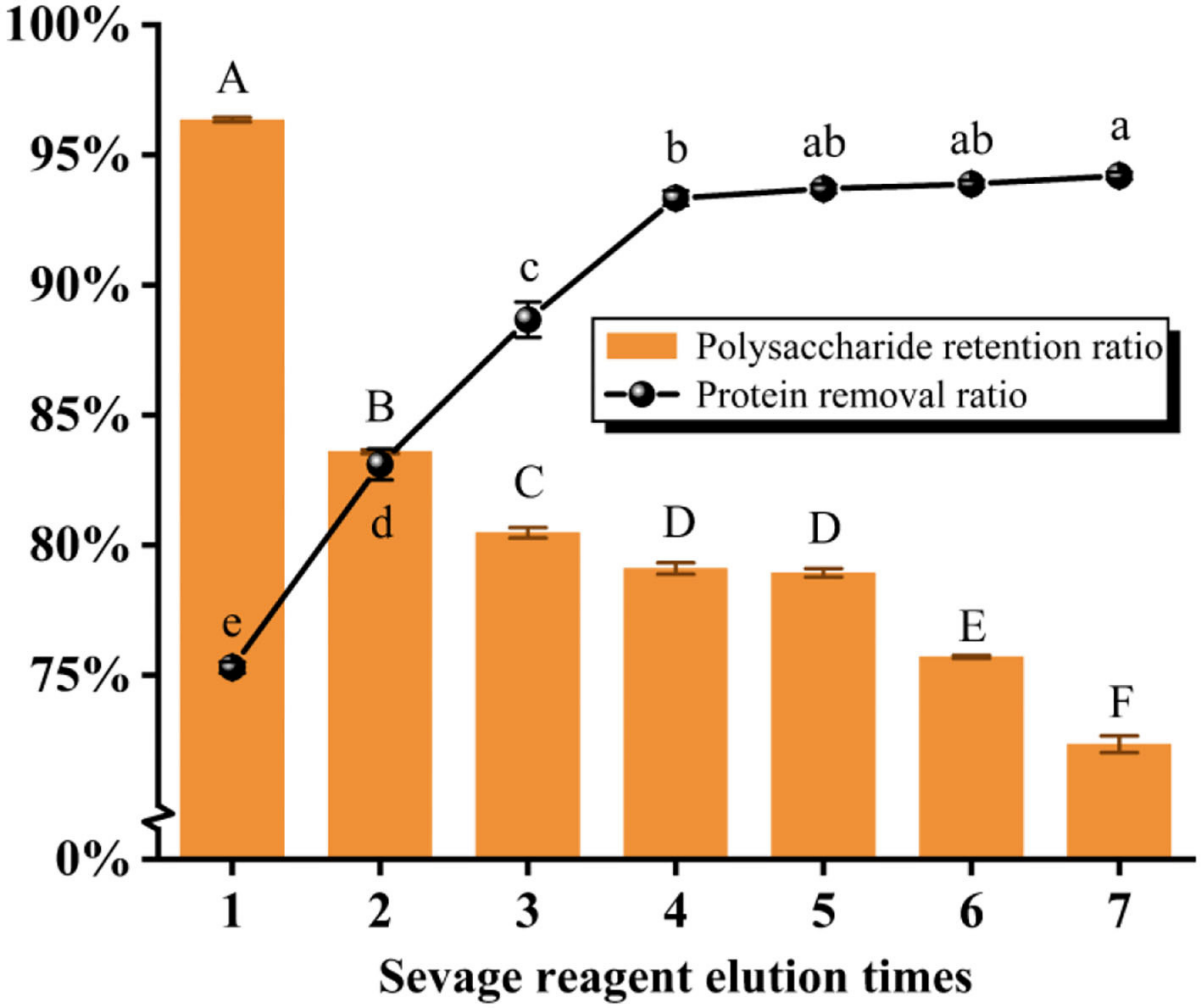
The results of elution times by the Sevage method. The same group of lowercase (uppercase) letters, if containing an identical letter is not a significant difference, whereas no identical letter is a significant difference (*P* < 0.05).

As presented in Figure 2 and Table 1, the protein removal ratio and the polysaccharide retention ratio presented an inverse relationship with the increase in the washing number by the Sevage method for 7 elutions. The protein removal rate gradually increased with the increase of the number of elutions from one to four times (*P* < 0.05), and the protein removal rate did not change significantly from five to seven times (*P* > 0.05), which combined with the literature (Ashurov et al., 2021) shows that the protein in the polysaccharide removed by the Sevage method is free protein and unable to eliminate the bound protein. After assessing the comprehensive scoring figures, the number of times of deproteinization by the Sevage method was finally chosen as five times. The subsequent isolation and purification processes were the same as in 2.2, to obtain PFSI-2 whose polysaccharide yield was 83.35 ± 0.52%.

**Table 1:** The results of elution times by the Sevage method.

**Sevage reagent elution times**	**Protein removal ratio**	**Polysaccharide retention ratio**	**Overall score**
1	75.29% ± 0.21%^e^	96.33% ± 0.09%^A^	85.81.% ± 0.09%^b^
2	83.10% ± 0.59%^d^	83.52% ± 0.08%^B^	83.31% ± 0.33%^e^
3	88.66% ± 0.68%^c^	80.40% ± 0.20%^C^	84.53% ± 0.19%^d^
4	93.34% ± 0.28%^b^	79.01% ± 0.22%^D^	86.17% ± 0.24%^ab^
5	93.69% ± 0.17%^ab^	78.85% ± 0.16%^D^	86.27% ± 0.17%^a^
6	93.88% ± 0.14%^ab^	75.60% ± 0.06%^E^	84.74% ± 0.06%^c^
7	94.20% ± 0.15%^a^	72.24% ± 0.32%^F^	83.22% ± 0.06%^e^

The superscript lowercase and uppercase letters indicates the same group of lowercase (uppercase) letters, if containing an identical letter is not a significant difference, where no identical letter is a significant difference (*P* < 0.05).

#### Deproteinization by the papain method

**Figure 3:**
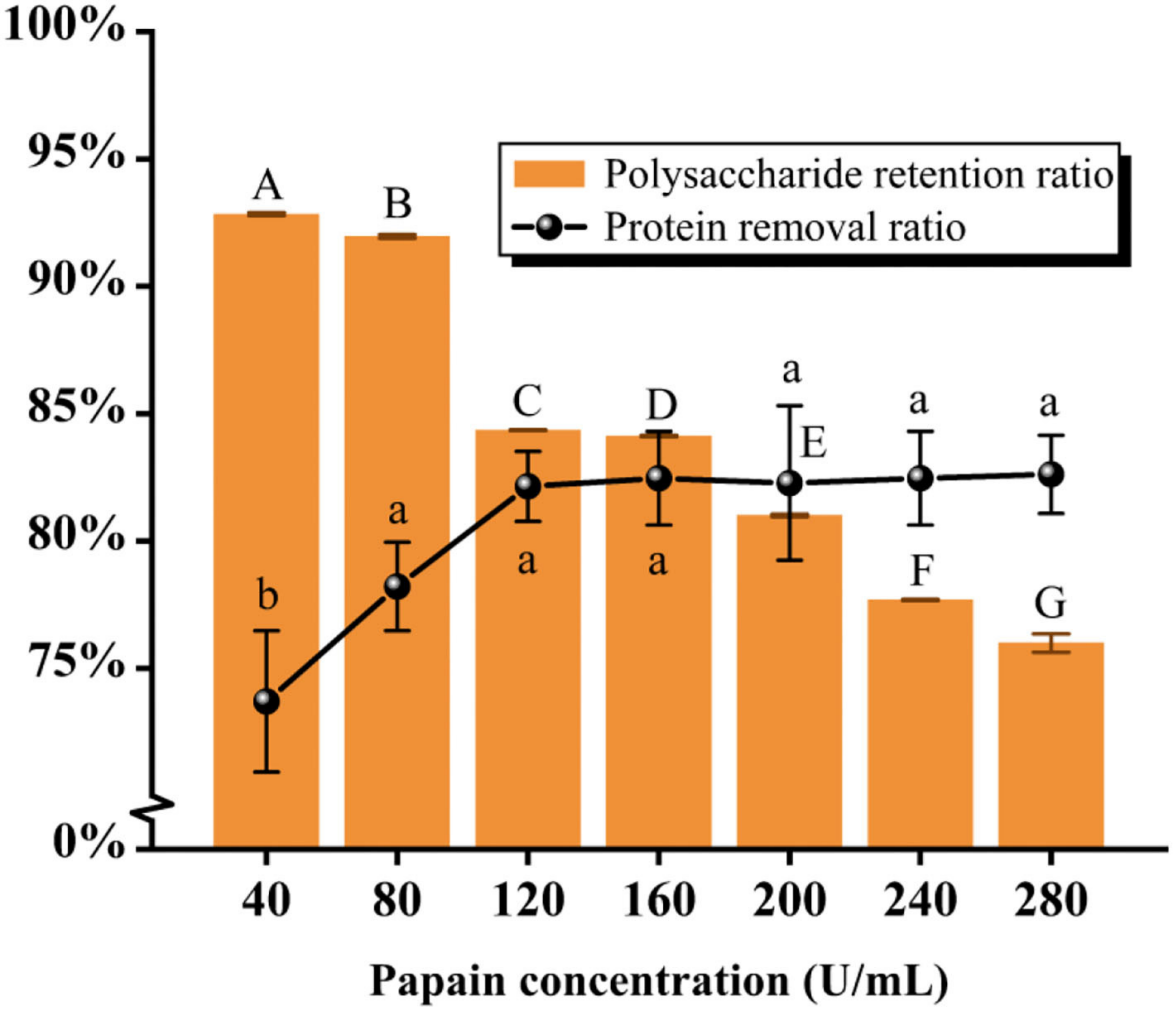
The results of elution times by the papain method. The same group of lowercase (uppercase) letters, if containing an identical letter is not a significant difference, whereas no identical letter is a significant difference (*P* < 0.05).

As seen in Figure 3 and Table 2, the results showed that the protein removal ratio, as well as the retention of polysaccharides, were inversely proportional to the increase of papain dynamics. Beyond the concentration of 80 U/mL, the protein removal ratio had no significant change with the accumulation of concentration (*P* > 0.05), which is because papain is an exclusive enzyme that only elutes the corresponding protein. The concentration of 80 U/mL papain was finally selected for protein removal by aggregating the scoring data. The subsequent isolation and purification process were the same as 2.2, yielding PFSI-3 whose polysaccharide yield was 83.62 ± 1.52%.

**Table 2:** The results of elution times by the papain method.

**Papain concentration**	**Protein removal ratio**	**Polysaccharide retention ratio**	**Overall score**
40 U/mL	73.70% ± 2.77%^b^	92.85% ± 0.06%^A^	83.28% ± 1.42%^ab^
80 U/mL	78.21% ± 1.74%^a^	91.99% ± 0.07%^B^	85.10% ± 0.91%^a^
120 U/mL	82.14% ± 1.37%^a^	84.40% ± 0.02%^C^	83.27% ± 0.67%^ab^
160 U/mL	82.46% ± 1.84%^a^	84.17% ± 0.03%^D^	83.32% ± 0.90%^ab^
200 U/mL	82.28% ± 3.04%^a^	81.06% ± 0.05%^E^	81.67% ± 1.48%^bc^
240 U/mL	82.46% ± 1.84%^a^	77.75% ± 0.03%^F^	80.11% ± 0.09%^cd^
280 U/mL	82.62% ± 1.54%^a^	76.07% ± 0.36%^G^	79.44% ± 0.76%^d^

The superscript lowercase and uppercase letters indicates the same group of lowercase (uppercase) letters, if containing an identical letter is not a significant difference, where no identical letter is a significant difference (*P* < 0.05).

#### Deproteinization by the TCA method

**Figure 4:**
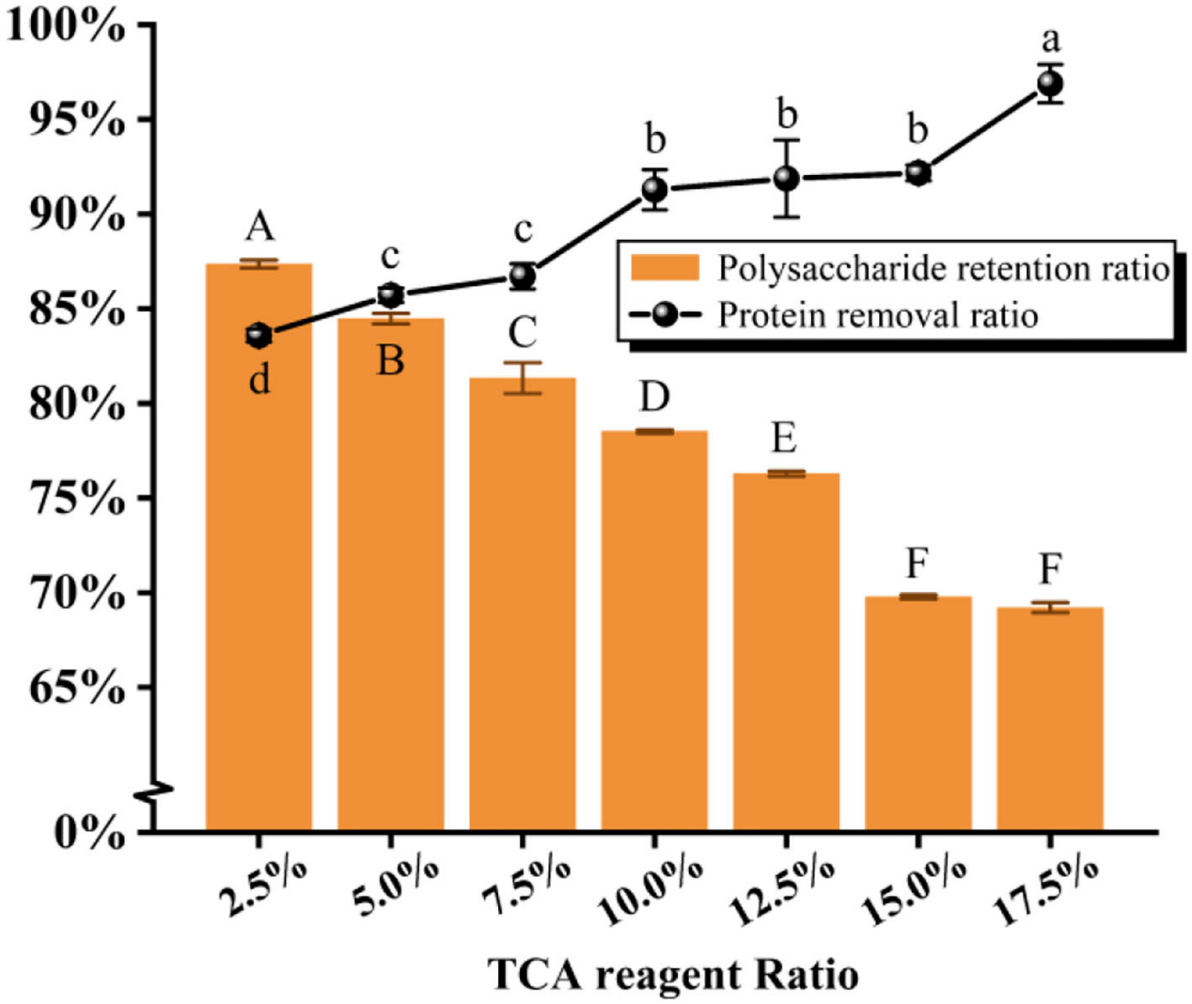
The results of elution times by the TCA method. The same group of lowercase (uppercase) letters, if containing an identical letter is not a significant difference, whereas no identical letter is a significant difference (*P* < 0.05).

As illustrated in Figure 4 and Table 3, it was found that the protein removal ratio, along with the retention of polysaccharides, decreased inversely with the increasing TCA ratio. From 10 to 15% of the TCA ratio, there was no significant change in protein removal ratio (*P* > 0.05), but at 17.5% ratio, a significant change in protein removal ratio was detected (*P* < 0.05), which was due to the violent reaction of the TCA method, generating somewhat reactive changes in the polysaccharide structure. By combining the scoring data, the 2.5% TCA was finally chosen for the deliquescence of proteins. The follow-up isolation and purification operation were identical to the above and led to PFSI-4 whose polysaccharide yield was 83.39 ± 0.64%.

**Table 3:** The results of elution times by the TCA method.

**TCA reagent ratio**	**Protein removal ratio**	**Polysaccharide retention ratio**	**Overall score**
2.5%	83.58% ± 0.34%^d^	87.34% ± 0.21%^A^	85.46.% ± 0.03%^a^
5.0%	85.71% ± 0.38%^c^	84.45% ± 0.27%^B^	85.08% ± 0.22%^a^
7.5%	86.70% ± 0.67%^c^	81.31% ± 0.81%^C^	84.00% ± 0.53%^b^
10.0%	91.87% ± 2.04%^b^	78.48% ± 0.09%^D^	84.78% ± 0.70%^ab^
12.5%	91.28% ± 1.06%^b^	76.24% ± 0.13%^E^	83.76% ± 0.44%^b^
15.0%	92.17% ± 0.42%^b^	69.74% ± 0.11%^F^	80.96% ± 0.19%^c^
17.5%	96.89% ± 1.01%^a^	69.17% ± 0.27%^F^	82.71% ± 0.38%^d^

The superscript lowercase and uppercase letters indicates the same group of lowercase (uppercase) letters, if containing an identical letter is not a significant difference, where no identical letter is a significant difference (*P* < 0.05).

### The analysis of UV spectra

**Figure 5:**
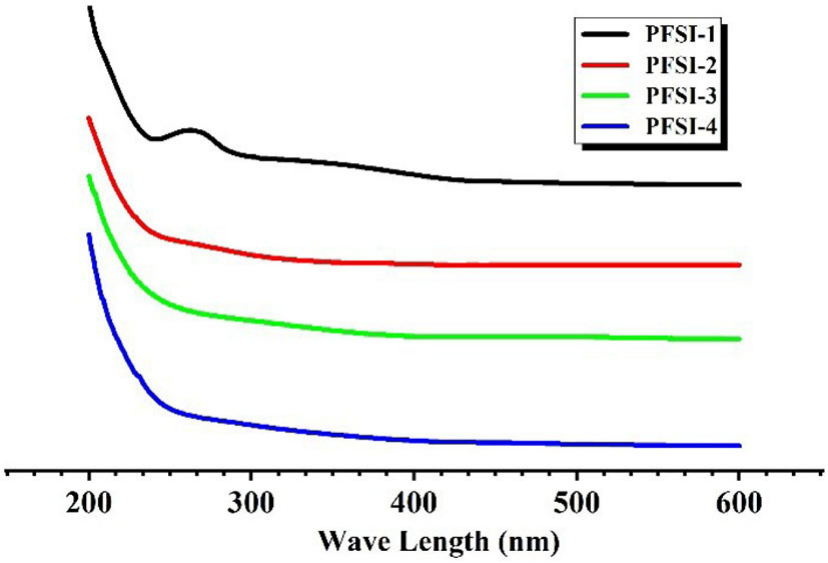
The results of UV spectra of PFSI-1, PFSI-2, PFSI-3, and PFSI-4.

The absence of obvious absorption peaks at 260 nm and 280 nm in Figure 5 indicates that PFSI-2, PFSI-3, and PFSI-4 do not contain impurity components such as nucleic acids and proteins, but the absorption peak is obvious around 260 nm in PFSI-1 which is not purified by deproteinization.

### The analysis of IR

**Figure 6:**
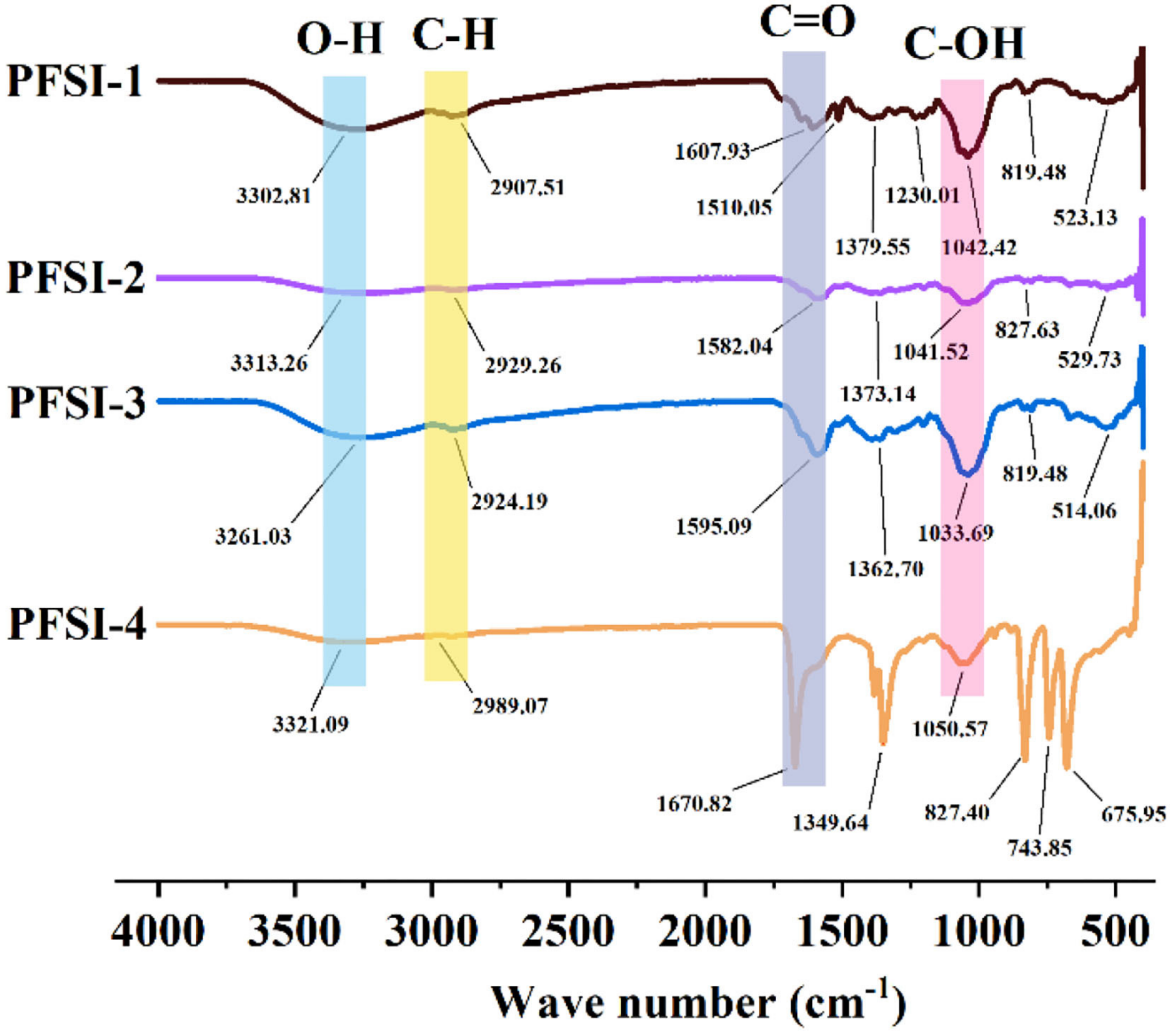
The results of infrared spectra of PFSI-1, PFSI-2, PFSI-3, and PFSI-4.

The IR spectra of PFSI-1, PFSI-2, PFSI-3, and PFSI-4 are illustrated in Figure 6. The peaks in the range of 3,600~3,000 cm^−1^, 2,900 cm^−1^, 1,600~1,400 cm^−1^, and 1,200~700 cm^−1^ are all typical polysaccharide absorption peaks. Among them, the absorption peaks in the range of 3,600~3,000 cm^−1^ suggest the possible presence of polysaccharides. The broad and strong absorption peak is the O-H functionality absorption peak, where the absorption peak is created by the hydroxyl group on the glycan chain. The waveforms of the peaks are wide and the wave numbers move to lower frequencies because of the presence of a large number of hydroxyl groups on the polysaccharide chain and the formation of a hydrogen bond within or between the molecules. The fainter absorption peak around 2,900 cm^−1^ is caused by the C-H stretching vibration. The more pointed absorption peaks around 1,600 cm^−1^ are (carboxyl or acid amine groups) C=O stretching vibrations and N-H variable angle vibrations, denoting absorption peaks caused by asymmetric carbonyl stretching. In combination with the UV spectrum, the absorption peaks occurred due to the stretching vibration of the carboxylate ion (–COO–) in the samples. The absorption peaks at around 1,400–1,200 cm^−1^ are attributed to the stretching vibration of C=C. The larger absorption peak between 1,200 and 1,000 cm^−1^ arises from the C-OH stretching vibration (Xiang et al., 2014). In this way, the characteristic absorption peaks at around 800 cm^−1^ are not only characteristic of α-type differential isomers but also caused by the stretching vibration of C=O on the pyranose ring, signifying that the glycan chain of the sample contains a pyranose ring structure, confirming the presence of a-D-pyranose (C_1_-H) as well as α and β-configuration glycosidic bonds. From the analysis of each absorption peak before 1,000 cm^−1^, it was initially inferred that the tested samples were saccharide structures. The results of the IR spectra show that PFSI-1, PFSI-2, PFSI-3, and PFSI-4 have the functional groups of polysaccharides as typical polysaccharides.

### The analysis of physicochemical properties of polysaccharides

From Table 4, it can be seen that the Molisch reaction was positive (+), indicating a violet ring appears at the junction of the two liquids as well as the presence of polysaccharides in the samples. The DNS reactions of PFSI-1, PFSI-2, PFSI-3, and PFSI-4 were positive (+), indicating the products of the reaction being brownish red and the presence of reducing polysaccharide in the samples.

**Table 4:** Physicochemical properties of PFSI.

**Testing indicators**	**PFSI-1**	**PFSI-2**	**PFSI-3**	**PFSI-4**
Molisch experiment	+	+	+	+
DNS experiment	+	+	+	+

+, Positive.

### The analysis of different carbohydrate sources on *L.f*

**Figure 7:**
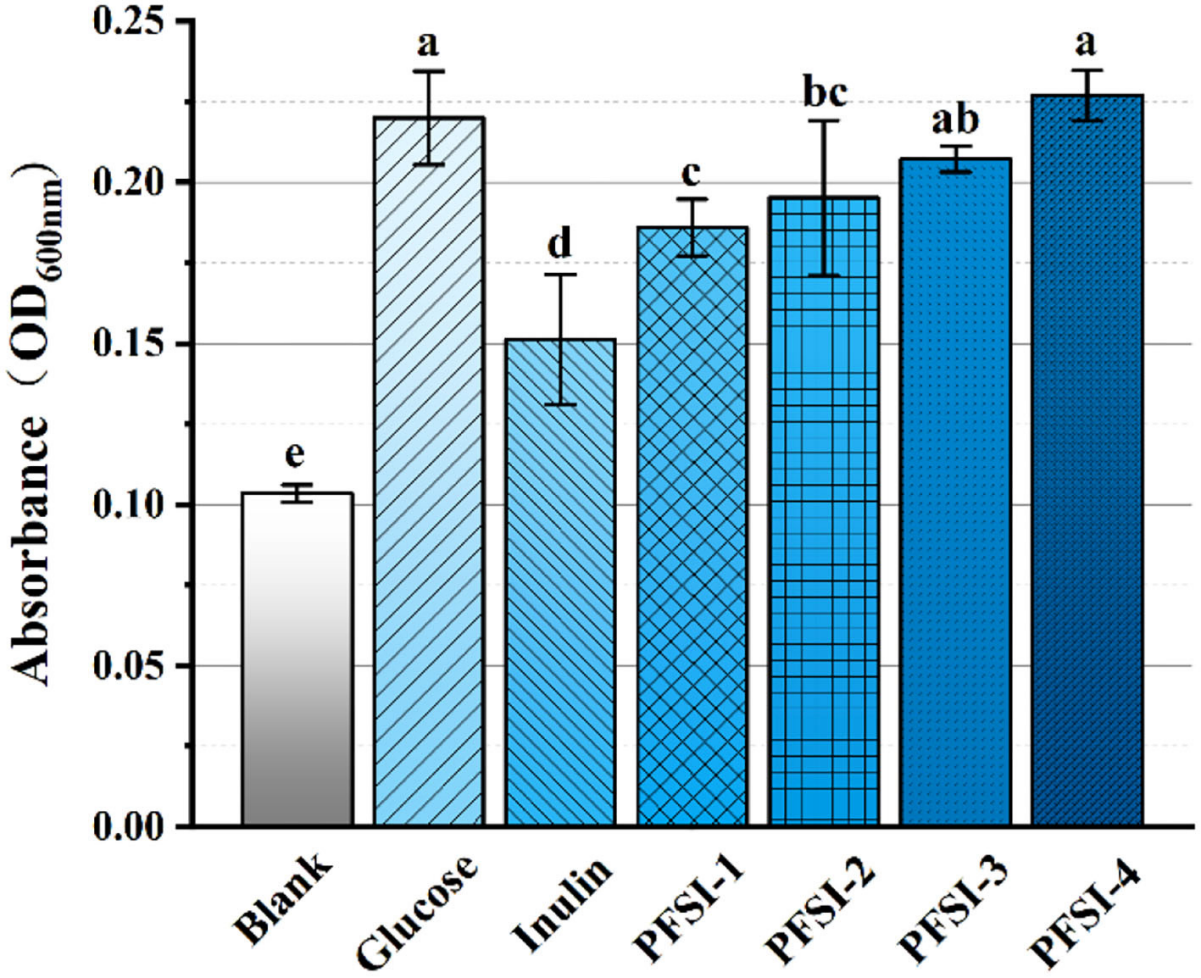
The results of infrared spectra of PFSI-1, PFSI-2, PFSI-3, and PFSI-4. The same group of lowercase letters, if containing an identical letter is not a significant difference, where there is no identical letter, is a significant difference (*P* < 0.05).

As demonstrated in Figure 7, each concentration of carbohydrate sources at 2.5 g/L had increased bacteriophage biomass vs. the blank control (*P* < 0.05). On the other hand, PFSI-4 was found with more bacteriophage biomass than PFSI-1, PFSI-2, and PFSI-3 (*P* < 0.05), which may be correlated with the molecular weight of polysaccharides, monosaccharide composition, and structure. When compared with glucose, the bacteriophage biomass grew with no significant difference (*P* > 0.05), but the aggregated amount was more than that of glucose. In conclusion, PFSI-4 can serve as the best available carbohydrate source for consequential studies.

### The analysis of the proliferative effect on *Lactobacillus* sp.

**Figure 8:**
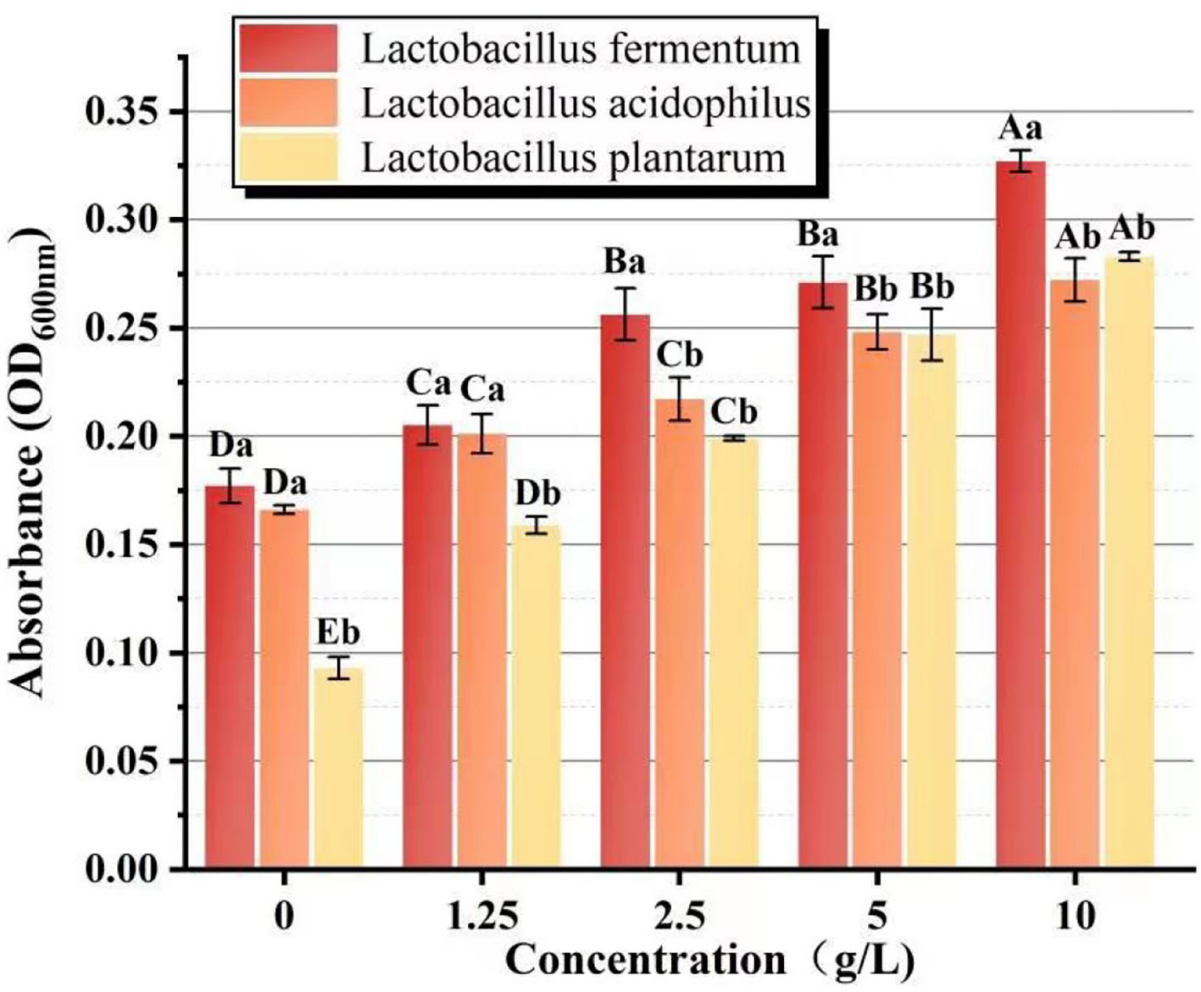
The comparison of the proliferative effect on *Lactobacillus* sp. Different capital letters indicate significant intra-group variability, *P* < 0.05; different lowercase letters indicate significant inter-group variability, *P* < 0.05.

The biomass of *L.f* , *L.a*, and *L.p* was found to increase significantly (P < 0.05) with increasing PFSI-4 concentration by the 2-fold dilution method. From Figure 8, it can be seen that to some extent it has a prebiotic function on Lactobacillus at 36 h, but the biomass, after acting on *L.f*, was more than that of *L.a* and *L.p*. This may suggest a selective and different intensity of role on various species of Lactobacillus. In a subsequent study, the viability associated with the action of PFSI-4 on *L.f* will be continued to be studied.

### The analysis of 36 h growth curve

**Figure 9:**
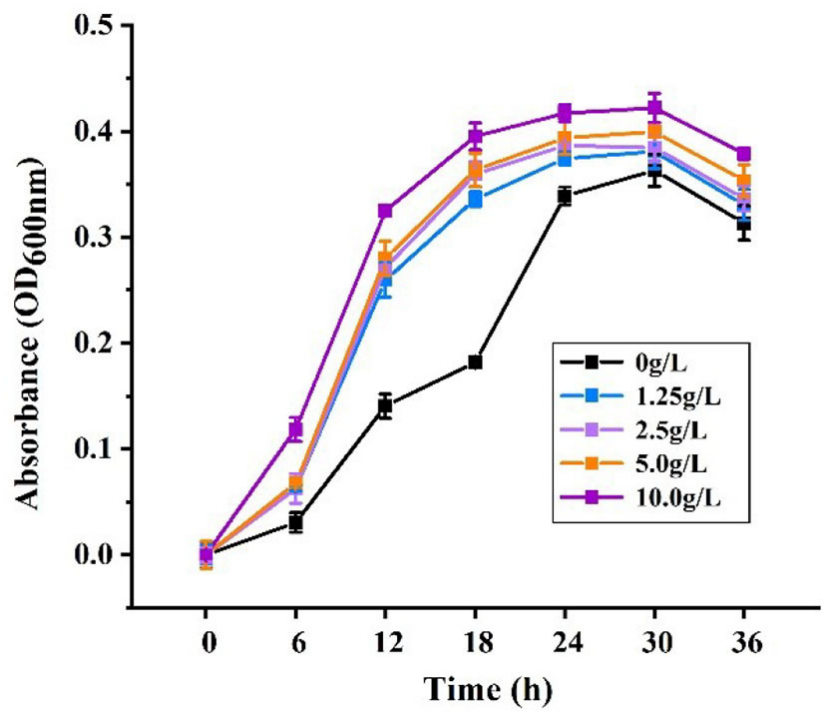
The effects of PFSI-4 on the growth curve of *L.f* within 36 h.

The results are shown in Figure 9, indicating that from 0 h to 30 h, PFSI-4 significantly accelerated the proliferation of *L.f* quantitatively (*P* < 0.05). Each concentration of PFSI-4 increased in the logarithmic growth period from 0 to 30 h, while the growth rate started decelerating or even decaying from 30 to 36 h. In summary, PFSI-4 could shorten the delay period of *L.f* culture, making it enter the logarithmic phase earlier, and accelerate the growth rate of logarithmic phase bacteria.

### The analysis of reducing saccharide content

**Figure 10:**
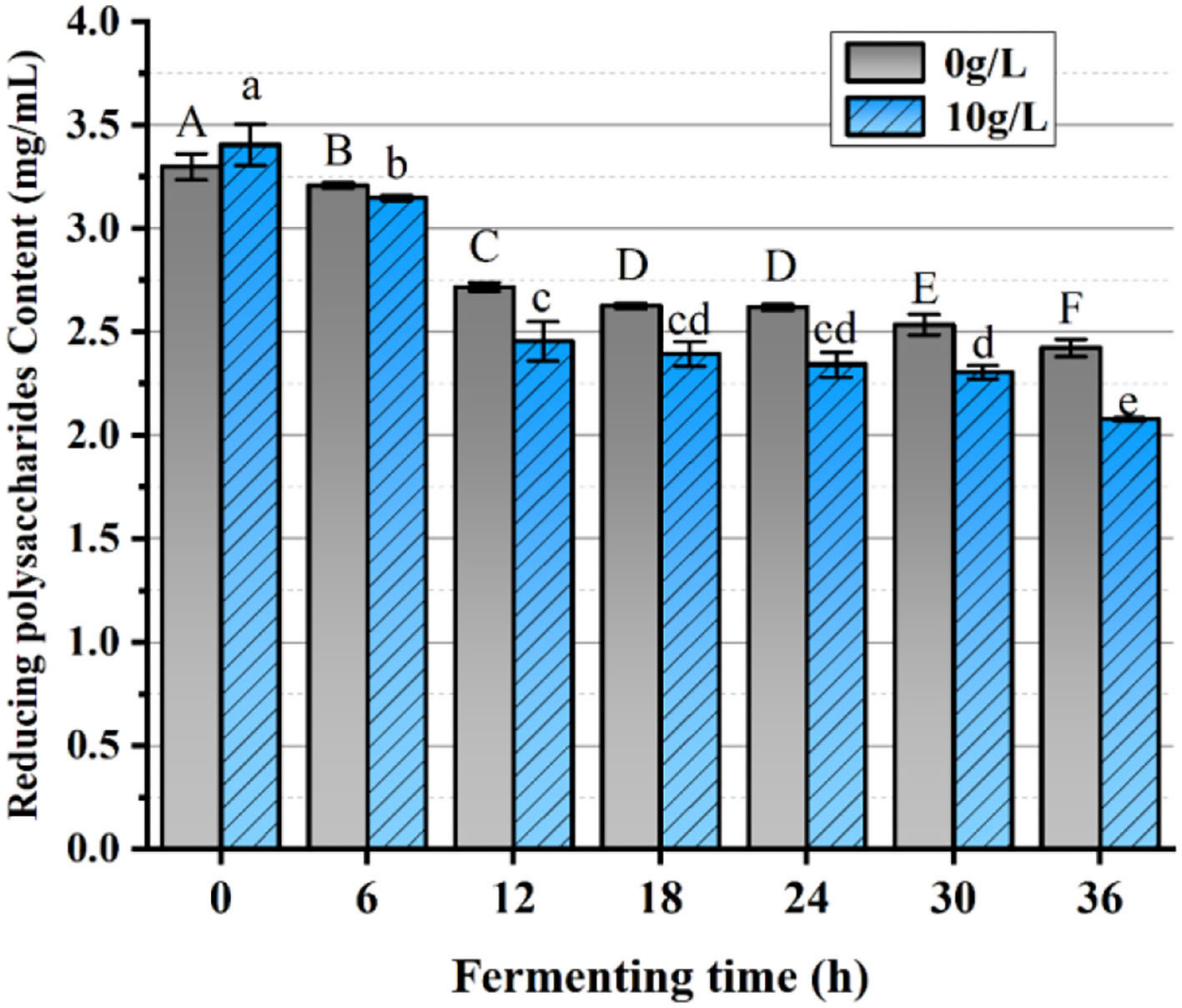
The variation of reducing saccharides content. The same group of lowercase (uppercase) letters, if containing an identical letter is not a significant difference, where no identical letter is a significant difference (*P* < 0.05).

The impact of PFSI-4 on *L.f* toward the utilization of reducing saccharides is plotted in Figure 10. The cultures with or without the addition of PFSI-4 had a temporal effect from 0 to 18 h. When *L.f* gradually entered the logarithmic phase, *L.f* growth was accelerated, and utilization of reducing saccharide was quickened. From 18 h to 30 h, as the *L.f* growth rate gradually leveled off, reducing saccharide utilization also gradually made no significant difference (*p* > 0.05). At 36 h, although the growth rate of *L.f* had gradually slowed down, reducing saccharide utilization continued to increase, though the rate at which it was increasing dropped. In the above, the reducing saccharide utilization in the experimental group with the addition of PFSI-4 was significantly higher than that in the blank control group, indicating that the addition of PFSI-4 could promote the absorption and utilization of reducing saccharides by *L.f* (*P* < 0.05) and facilitate its growth.

### The analysis of protein content

**Figure 11:**
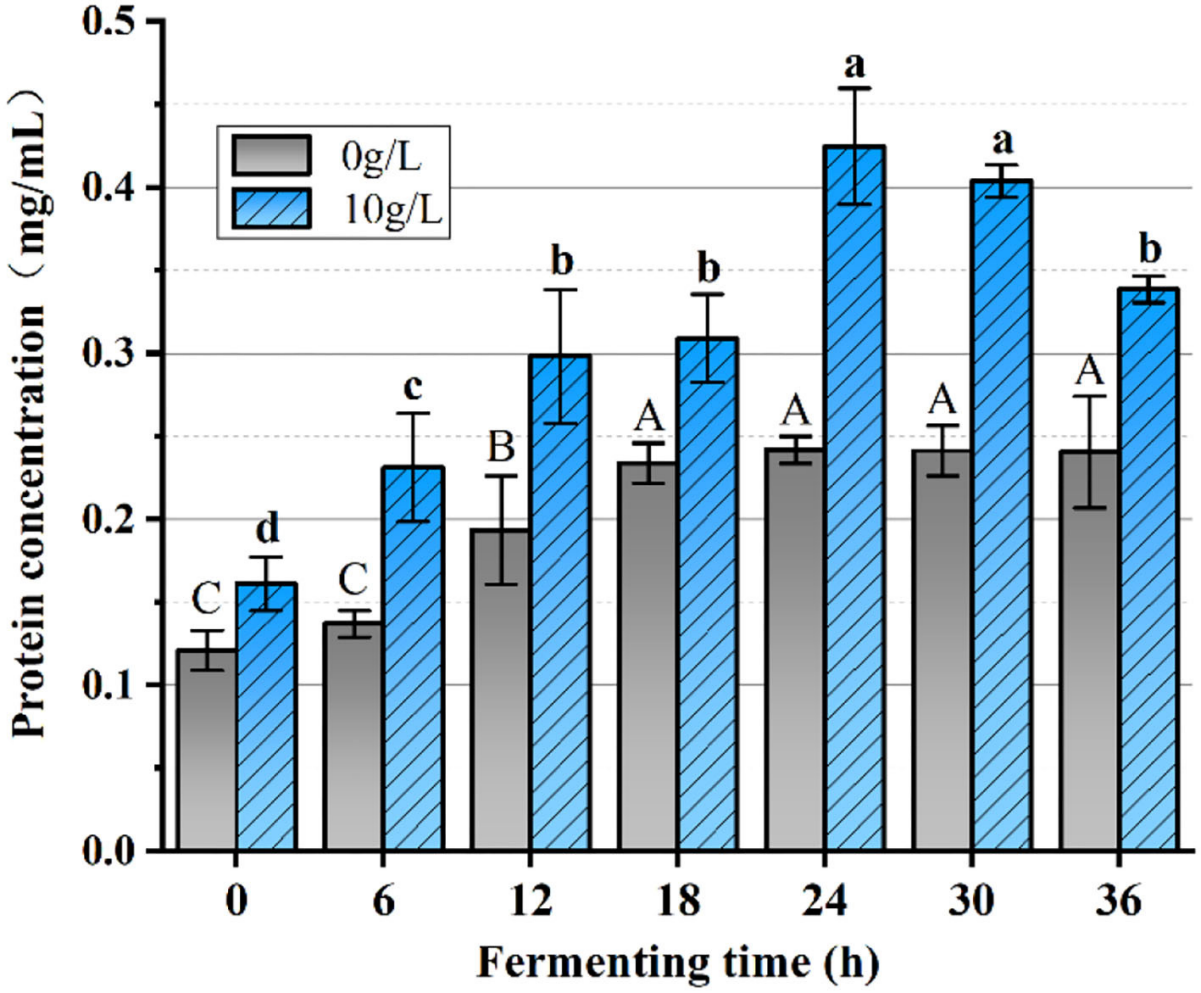
The variation of protein content. The same group of lowercase (uppercase) letters, if containing an identical letter is not a significant difference, where no identical letter is a significant difference (*P* < 0.05).

The effect of PFSI-4 on protein synthesis is shown in Figure 11. The protein content at all stages of growth was higher than that without PFSI-4, indicating that PFSI-4 could promote *L.f* protein synthesis (*P* < 0.05) and improve the physiological activeness of the organism.

### The analysis of lactate content

**Figure 12:**
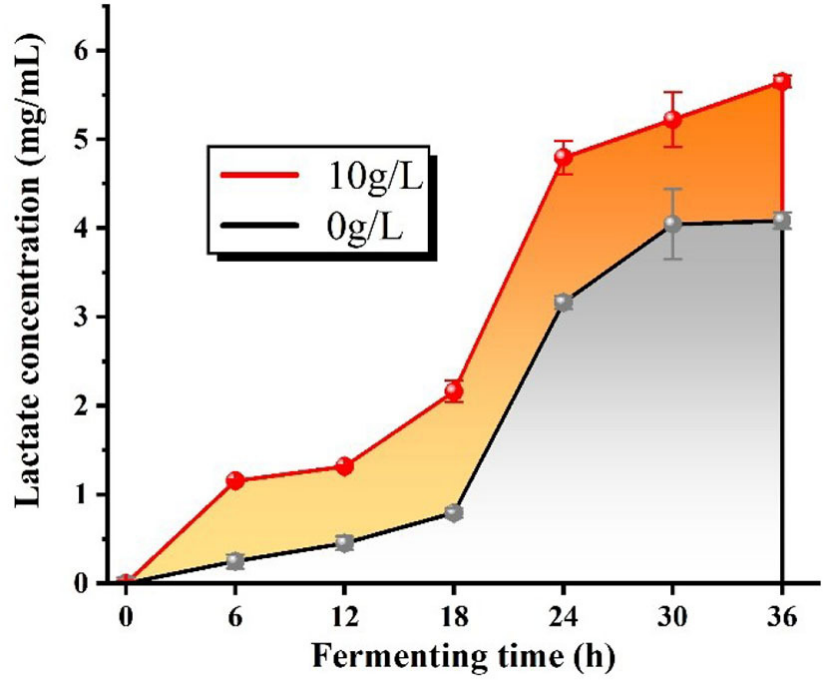
The variation of lactate content.

Producing acid ability is an essential physiological characteristic of lactobacilli, and lactate is the major metabolite of organoleptic acid in *L.f* . The changes in lactate content in the fermentation broth reflected the growth status of the bacteria, and the lactate synthesis velocity was enhanced when the bacteria grew faster. From Figure 12, we can conclude that the lactate concentration gradually increased with the increase of fermentation time with or without the addition of PFSI-4, but the lactate content of each culture section with the addition of PFSI-4 was always superior to that without the addition of PFSI-4, which means that PFSI-4 promoted the growth of *L.f* and increased the lactate content (*P* < 0.05).

### The analysis of the dynamics of LDH, SOD, and GSH-Px

**Figure 13:**
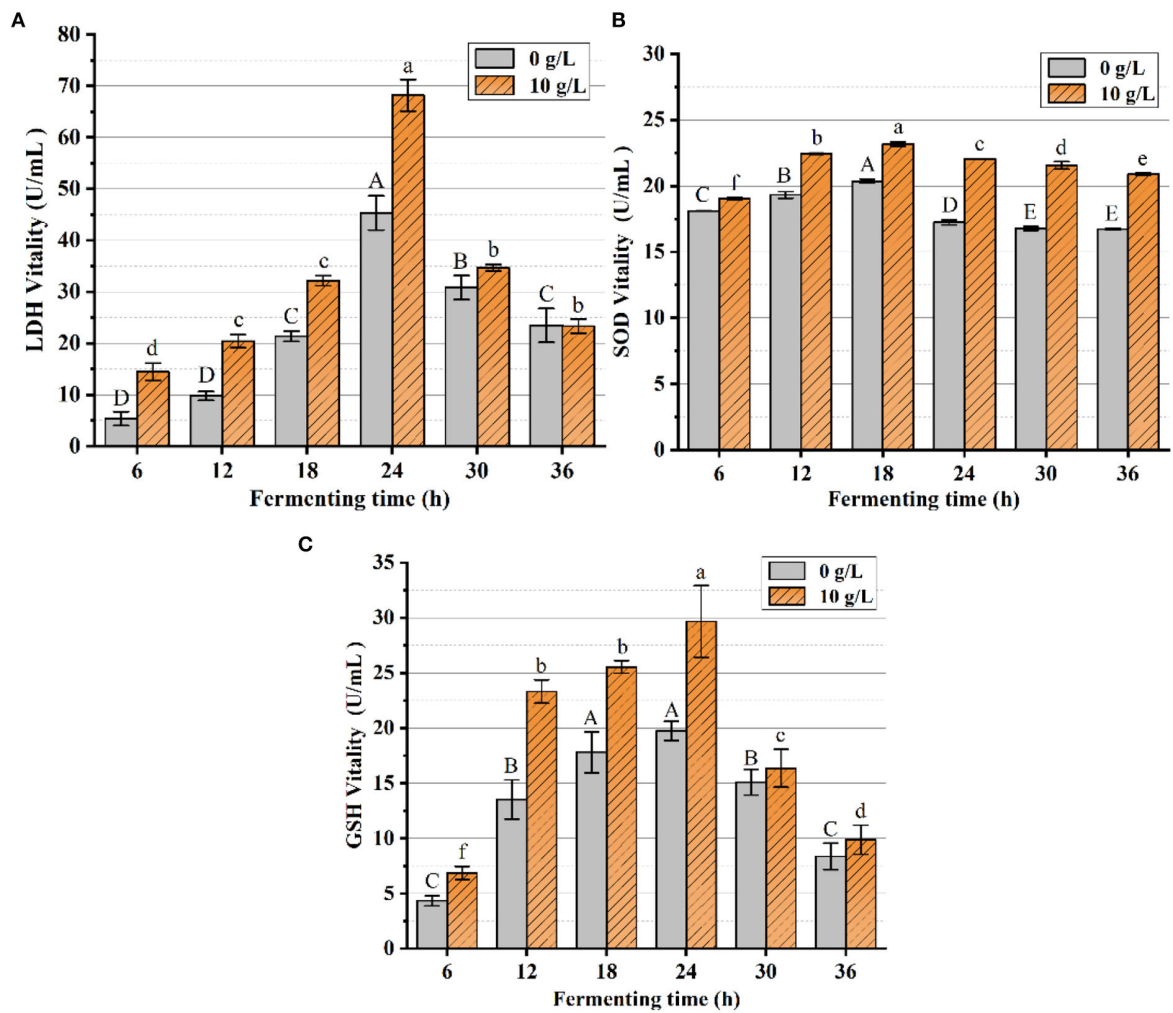
The variation of LDH, SOD, and GSH-Px contents. In the figure, **(A)** is the variation of LDH content, **(B)** is the variation of SOD content, and **(C)** is the variation of GSH-Px content. The same group of lowercase (uppercase) letters, if containing an identical letter is not a significant difference, where no identical letter is a significant difference (*P* < 0.05).

As shown in Figure 13A, LDH catalyzed the conversion of pyruvate to lactate under anaerobic conditions, and the LDH vitality was markedly elevated by the additive PFSI-4 vs. the non-additive carbohydrate source. At 24 h fermentation time, the LDH viability of the experimentally administered group reached approximately the maximum of 68.19 U/mL, and the LDH viability of the blank control group reached approximately the maximum of 45.33 U/mL. PFSI-4 remarkably boosts the viability of LDH (P < 0.05).

From Figures 13B,C, it was observed that SOD and GSH-Px vitality changes achieved maximum vitality at about 18 h. The SOD and GSH-Px vitality of the experimental group was superior to that of the control group at all measured time points. SOD and GSH-Px have been identified as critical antioxidant enzymes in cells, which scale off excess free radicals and defend cells from damage and assume a vital role in the oxidative and antioxidant balance of the organism. The level of SOD and GSH-Px activity under anaerobic conditions represents the strength of the physiological activity of the cells. The addition of PFSI-4 increased the activity of LDH, SOD, and GSH-Px, indicating that PFSI-4 could strengthen the physiological activity of *L.f* (*P* < 0.05) and stimulate its growth.

## Discussion

*L.f* is one of the common species of Lactobacilli in food, with a relatively high safety profile and the unique property that it can tolerate, colonize, and survive under harsh conditions (Ali et al., 2018). For this study, *L.f* (GDMCC 1.1796) was isolated from fermented milk. The strain itself is commonly used in food fermentation to produce extracellular polysaccharides (EPS). It is well-known that L.f is a probiotic with cholesterol-lowering properties (Ali et al., 2020), and its functional properties and the contribution of its application to human health have become a hot topic of research. Some scholars (Yang et al., 2021) have evaluated the effectiveness of *L.f* (ZJUIDS06) as a probiotic in hypercholesteremic golden hamsters. It revealed that *L.f* (ZJUIDS06) supplementation for 8 weeks increased colonic SCFA levels (P < 0.05), decreased serum LDL, total cholesterol, and triglyceride levels, and caused changes in the cecum microbiota of the hamsters. Notably, oral administration of *L.f* (ZJUIDS06) increased the relative abundance of *L.f* parapsilosis in the cecum, which acts as a biomarker for colonic SCFA production and improvement of serum cholesterol levels. Altogether, *L.f* (ZJUIDS06) ameliorated hyperlipidemia in the hamsters, which correlated with increased SCFA levels and relative abundance of paramecium-like substances, suggesting its potential importance in functional foods that could help lower cholesterol. Besides, it was also found (Gawande et al., 2021) that exopolysaccharide (EPS400) was isolated from *L.f* (LFNCDC400). Under gastrointestinal conditions, EPS400 demonstrates a greater reduction in cholesterol bio-accessibility than LFNCDC400. In experimental animal studies, a known cholesterol-lowering β-glucan and EPS400 significantly reduce and maintained cholesterol status in hypercholesterolemic rats compared to LFNCDC400 (*p* < 0.05). EPS400 was associated with a significant reduction and maintenance of cholesterol status in hypercholesterolemic rats compared to LFNCDC400. Moreover, the pro-life properties of *L.f* are not only cholesterol-lowering but also have other active functions. *L.f* (CECT5716) was fortified with infant formula and fed to infants from 1–6 months of age. This strain improved infant health and reduced the incidence of gastrointestinal infections (Gil-Camposa et al., 2012). In terms of maintaining intestinal health, it was proven through *in vivo* tests that *L.f* (CECT5716) could prevent and alleviate intestinal damage (Olivares et al., 2007). Therefore, *L.f* itself has some potential activity, and the effective development of its EPS and the exploration of different aspects of its characteristics will be one of the important ways to improve the health of all human beings.

Polysaccharides, in current studies, exist mainly in two forms, as pure sugar chains and as sugar-protein combinations to form glycopeptides or glycoproteins. PFSI is a heteropolysaccharide, and its activity against several disorders may be the glycogen component of the crude polysaccharide, the protein component of it, or the combination of the glycogen and protein. Each of the distinct deproteinization processes resulted in different activities (Xiang et al., 2014; Mu et al., 2022); (Wang et al., 2022) that were required for the activity requirements derived from the products, and hence it is necessary to discuss them categorically. The conventional Sevage method, had a relatively high-quality deproteinization rate, but caused a lot of polysaccharide loss due to repeated iterations and wasted a large amount of organic solvent, but the activity remained comparatively sound. Since the proteins removed by the Sevage method were mostly free proteins, the glycoconjugate proteins were not destroyed. The papain method enzymatically degraded the glycoconjugate proteins and destroyed them, thus reducing the biological inactivity of PFSI to some extent. The TCA method had higher destruction of polysaccharide activity under drastic conditions, so its potential for a variety of activities was increased by certain structural changes (Liang et al., 2021).

In this paper, we investigated the proliferative *L.f* activity of PFSI obtained by isolation and purification without deproteinization and three different deproteinizations through synergistic extraction of PFSI with ultrasound and microwave, and they all could potentiate the effect of *L.f* proliferation. It indicates that PFSI with or without deproteinization has a proliferative function on *L.f* activity. However, through different deproteinization methods, and by comparing the probiotic activity of the positive control group, it was found that the probiotic activity of PFSI purified by deproteinization using the TCA method was better. It is possible that the TCA method can be effectively used to remove the proteins affecting the probiotic activity, but we cannot exclude that it may be connected with the degree of polysaccharide purification, molecular weight, molecular structure, and so on. This suggests that the protein contained in PFSI affects the probiotic viability of PFSI to *L.f* to some extent. Therefore, when the probiotic viability of polysaccharides is explored, we suggest the processing of deproteinization and different polysaccharides contain different structural proteins and free protein components, which can be categorized and explored and the best way to deproteinize the obtained polysaccharides. The TCA method of deproteinization can be considered the best choice when removing protein is necessary to maintain the peptide's biological activity.

## Conclusion

After eluting PFSI by the ultrasonic microwave method, the optimal process conditions for protein removal from PFSI is five times elution through the Sevage method, 80 U/mL concentration of papain, and 2.5% concentration of TCA, respectively. All four PFSI were capable of promoting *L.f* proliferation, and we found that PFSI-4 had both quantitative and chronological effects on *L.f* ; and the addition of 10 g/L PFSI could promote the utilization of reducing sugars and the synthesis of protein and lactate in the bacterium, as well as boost the biomass of *L.f* and meanwhile the viabilities of LDH, SOD, and GSH-Px. It is found that the TCA method could effectively remove the proteins affecting the probiotic properties in PFSI-4, and PFSI could improve the physiological function and facilitate the growth of *L.f* . Furthermore, UV and IR spectra verified that the four PFSIs were typical polysaccharide structures and that the proliferation of *L.f* was better when PFSI-4 was applied to *L.f* , *L.a*, and *L.p* in comparison. PFSI may be a potentially beneficial prebiotic and intestinal immune enhancer, and the results of this study on the development of functional foods with probiotic benefits provide a scientific ground for broadening its application in the relevant food fields. Additionally, it serves to provide a scientific basis for the exploitation of Lactobacillus polysaccharide complex products, which could provide a theoretical reference for future research on prebiotics and probiotics.

## Data availability statement

The raw data supporting the conclusions of this article will be made available by the authors, without undue reservation.

## Author contributions

Conceptualization: WZ. Methodology and validation: CY, WZ, and YZ. Software, data curation, and writing—original draft: WZ and DY. Formal analysis: CY and WZ. Investigation: WZ and YZ. Resources: DY. Writing—review and editing: CY, WZ, and DY. All authors have read and agreed to the published version of the manuscript.

## Funding

We are grateful for the financial support from the Ph.D. Enhancement Program at Zhuhai College of Science and Technology.

## Conflict of interest

The authors declare that the research was conducted in the absence of any commercial or financial relationships that could be construed as a potential conflict of interest.

## Publisher's note

All claims expressed in this article are solely those of the authors and do not necessarily represent those of their affiliated organizations, or those of the publisher, the editors and the reviewers. Any product that may be evaluated in this article, or claim that may be made by its manufacturer, is not guaranteed or endorsed by the publisher.
